# Polypharmacy and pattern of medication use among patients with gastroesophageal reflux disease: results from Pars Cohort study

**DOI:** 10.1186/s12876-023-03086-7

**Published:** 2023-12-14

**Authors:** Arash Ghamar-Shooshtari, Zahra Rahimian, Hossein Poustchi, Zahra Mohammadi, Bita Mesgarpour, Mohammadreza Akbari, Alireza Kamalipour, Seyed Reza Abdipour-Mehrian, Elham-Sadat Hashemi, Pooria Zare, Kamran Bagheri Lankarani, Reza Malekzadeh, Fatemeh Malekzadeh, Hossein Molavi Vardanjani

**Affiliations:** 1 MD-MPH Department, School of Medicine, Shiraz University of Medical Sciences, Shiraz, Iran; 2 Ophtalmology Resident of Farabi Eye Hospital, Tehran University of Medical Sciences, Tehran, Iran; 3 Liver and Pancreatobiliary Disease Research Center, Digestive Disease Research Institute, Tehran University of Medical Sciences, Tehran, Iran; 4 Vice Chancellery for Research and Technology, National Institute for Medical Research and Development (NIMAD), Tehran, Iran; 5 Shiraz Nephro-Urology Research Center, Shiraz University of Medical Sciences, Shiraz, Iran; 6 Department of Ophthalmology, Shiley Eye Institute, Hamilton Glaucoma Center, University of California, San Diego, CA USA; 7 Health Policy Research Center, Institute of Health, Shiraz University of Medical Sciences, Shiraz, Iran; 8 Pathology Resident of Kermanshah University of Medical Sciences, Kermanshah, Iran; 9 Digestive Diseases Research Institute, Tehran University of Medical Sciences, Tehran, Iran; 10 Digestive Diseases Research Center, Digestive Diseases Research Institute, Shariati Hospital, Tehran University of Medical Sciences, Tehran, Iran; 11 Research Center for Traditional Medicine and History of Medicine, School of Medicine, Shiraz University of Medical Sciences, Shiraz, Iran

**Keywords:** Gastroesophageal reflux Disease, Polypharmacy, Medication use, Pharmacoepidemiology, Drug-drug interaction, Iran

## Abstract

**Background:**

Gastroesophageal Reflux Disease (GERD) is a common chronic condition. Its chronic nature may affect the pattern of medication use. This study aimed to investigate the prevalence, associated factors, and patterns of polypharmacy and medication use among GERD patients in southwestern Iran.

**Methods:**

We used data from the Pars Cohort Study. We classified drugs using the Anatomical Therapeutic Chemical classification system. The Lexicomp® database was used to assess potential drug-drug interactions. Multivariable Poisson regression was applied. Adjusted prevalence ratio (PR) and its 95% confidence interval (CI) were estimated.

**Results:**

A total of 9262 participants were included. Among 2,325 patients with GERD, age-standardized prevalence of polypharmacy was 9.5% (95% CI: 7.5%, 11.6%) in males, and 19.3% (95% CI: 17.2%, 21.4%) in females. The PR of experiencing Polypharmacy by GERD patients compared to non-GERD patients was 1.82 (95% CI: 1.61, 2.05%). Multimorbidity (PR: 3.33; CI: 2.66, 4.15), gender (PR: 1.68; CI: 1.30, 2.18), and metabolic syndrome (PR: 1.77; CI: 1.45, 2.15) were associated with polypharmacy among GERD patients. Drugs for acid-related disorders were the most common used drugs among men, women and elders. We found that 13.9%, 4.2%, and 1.1% of GERD patients had type C, D and X drug interactions, respectively.

**Conclusion:**

GERD is correlated with a higher prevalence of polypharmacy. Among GERD patients, females, those with multi-morbidities, and those with metabolic syndrome may be affected more by polypharmacy. Considering the fairly high rate of interactions identified, a review of the medication list is essential when approaching GERD patients, and physicians must check for medications that may worsen GERD.

**Supplementary Information:**

The online version contains supplementary material available at 10.1186/s12876-023-03086-7.

## Background

Gastroesophageal reflux disease (GERD) is on the rise worldwide, with roughly four-tenths of Americans experiencing its symptoms in the past and one-third in the past week [1, 2]. In Iran, the overall, monthly, weekly, and daily prevalence of GERD symptoms is 43.07%, 18.62%, 12.50%, and 5.64%, respectively [3]. This chronic disease causes troubling symptoms and complications, affecting patients’ well-being and quality of life [4, 5]. Esophageal ulcers, dysphagia, stricture, upper gastrointestinal bleeding, and Barrett’s esophagus are common complications, with extra-esophageal complications also possible [4, 5].

Polypharmacy is defined as using five or more drugs, affecting patients’ quality of life, well-being, and survival [6, 7]. Additionally, polypharmacy is a risk factor for adverse outcomes such as hospital admission, drug interactions, adverse drug side effects, and poor medication compliance, resulting in severe negative clinical outcomes [8, 9]. The pattern and prevalence of polypharmacy differ based on the patients’ age and culture, the healthcare system, and the timing of polypharmacy studies, with older people being more likely to use more medications [10, 11]. As some diseases prevalent in the elderly (e.g., diabetes, Parkinson’s disease, and Alzheimer’s disease) affect the esophagus and gastrointestinal tract, old patients are at higher risk of GERD [12]. Moreover, medications such as nitrates, benzodiazepines, anticholinergics, antidepressants, NSAIDs, and lidocaine exacerbate GERD. Although many OTC drugs are used for heartburn, they are associated with side effects. Histamine-2 receptor antagonists are avoided for patients with delirium, while proton pump inhibitors increase the risks of dementia, osteoporosis, and infections [13, 14]. Hence, it is crucial to appreciate the patterns of drug use among patients in different settings.

In contrast to developed countries, there is a dearth of data regarding polypharmacy in less developed countries [15]. Identifying the patients with polypharmacy risks helps deprescribing and reduce the number of potentially inappropriate medications, optimize the health benefits of patient’s medications, and reduce medication-related adverse events, particularly at an early stage [16]. Although drugs used for GERD are effective and mostly tolerable, studies indicate that they are often prescribed inappropriately [17–19]. Therefore, they can endanger the patients with increased risks of adverse drug reactions such as *Clostridium difficile* infection, hypomagnesemia, pneumonia, chronic kidney disease, and fractures [18]. This issue is more significant in older patients with multiple chronic diseases and polypharmacy [20].

In light of the mentioned points, we investigated the prevalence, patterns, and factors associated with polypharmacy among patients with GERD in a cohort of patients in Iran. Specifically, we sought to answer the following research questions: What is the prevalence of polypharmacy in patients with GERD compared to patients without GERD? What are the factors related to the prevalence of polypharmacy in GERD patients? What is the pattern of drug consumption in these patients? What is the prevalence of drug-drug interactions in these patients? Finally, which drugs are the most common ones involved in potential drug-drug interactions?

## Methods

### Study design, setting, and participants

This prospective cross-sectional study explored the population-based prevalence, patterns, and correlates of polypharmacy among GERD patients in Valashahr, southern Iran. Data from the Pars Cohort Study (PCS) were used as baseline data; 9,270 inhabitants of Valashahr, a semi-urban in the Fars province in the south of Iran, have been participating in this cohort since 2012. The PCS enrolled individuals aged 40 to 75 years to determine the epidemiology and risk factors of non-communicable diseases. This prospective population-based cohort’s study protocol has previously been published [21]. No sample size was calculated as this study was based on PCS baseline data.

### Data collection and variable definition

Data was collected by clinical history, physical examination, interview, measuring anthropometric indices, and biomedical samples. Skilled personnel who were trained medical doctors and standardized tools were involved in all cases. More details are available elsewhere [21].

Participants were asked about experiencing heartburn and acid regurgitation within the past year and frequencies of these symptoms were also inquired. GERD was defined as experiencing heartburn and/or acid regurgitation weekly or more frequent. The process of ruling out cardiac chest pain was conducted by medical doctors through an assessment of the patient’s clinical history and a physical examination.

Polypharmacy was defined as using five or more drugs simultaneously [6]. Patients were asked to bring their bag of medications, and a nurse listed the drugs and ensured that the patients were using all the medications inside their bag. Patients were categorized into polypharmacy (using five or more drugs) and non-polypharmacy (using fewer than five drugs) groups based on the number of drugs they were using concurrently. We used the first level of the Anatomical Therapeutic Chemical (ATC) classification system [22] to categorize drugs other than complementary and alternative medicines. Also, to categorize alimentary tract and metabolism drugs, ATC code A was used. To check for drug-drug interactions, we processed the raw data on the drugs by removing duplicates and correcting all misspelled drug names. Then, potential drug-drug interactions were identified using the Lexicomp® database and classified by its risk rating system, in which the clinical significance of interactions raised as we progress from type A and B (safe) to type C (monitor therapy), type D (modify regimen), and type X interactions (avoid combination) [23].

We extracted the potential covariates of polypharmacy from the PCS database and analyzed them. These covariates were age (< 50, 50–59, and ≥ 60), gender (male and female), education (illiterate and literate), ethnicity (Fars and non-Fars), marital status (married, divorced/widowed), socioeconomic status (low, low-middle, middle-high, and high), physical activity (low, moderate, and high), body mass index (< 25, 25–30, and > 30 kg/m2), metabolic syndrome (yes or no), alcohol use (no or ever use), cigarette smoking (no or ever smoking), tobacco use (yes or no), opium use (no or ever use), and comorbidities (one, two or three, more than four).

Participants’ socioeconomic status (SES) was measured through using their self-reported assets. Asset analysis was performed by multiple correspondence analysis, and a latent factor was estimated. Considering the quartiles of the estimated latent factor, participants were classified into four groups (low, low-middle, middle-high, and high). Metabolic syndrome was defined based on the criteria introduced by Alberti et al. [24] for Asian population. We obtained Physical activity data through International Physical Activity Questionnaire (IPAQ), then converted to Metabolic Equivalent of Task (MET) scores. In the next step, we categorized participants into three distinct groups including high (at least 3000 MET-minutes/week), moderate (at least 600 MET-minutes/week), and low (less than 600 MET-minutes/week).

### Statistical analysis

Mean, standard deviation (SD) and frequency were calculated to describe variables where appropriate. To assess the prevalence of polypharmacy and its 95% confidence interval (CIs), the Poisson distribution was used. The age-standardized prevalence (ASR) was estimated considering the world standard population [25](WHO 2000–2025). The chi-squared and Mann-Whitney U tests was used for univariate analyses to check the association of categorical variables with the polypharmacy Prevalence. To investigate the independent correlation of potential covariates with the polypharmacy prevalence, multivariable Poisson regression was used. Variables with univariate *P*-values less than 0.3 were candidates for inclusion in the multivariable modeling as potential correlates. To proportionate the final multivariable model, a backward elimination method was used. The adjusted prevalence ratios (PR) and its95% CIs were presented. A *P*-value less than 0.05 was considered statistically significant. Stata software (Release 11, College Station, TX: Stata Corp LLC) was used for data analysis.

## Results

Out of 9262 participants, 2,325 (25.1%) had at least weekly GERD symptoms, of whom 843 (36%) and 1482 (64%) were male and female, respectively (Table 1). The mean age of patients with GERD was 53.7 ± 10.1 (year). The prevalence of polypharmacy among patients with and without GERD was 15.6% (95% CI 14.2%, 17.2%) and 8.6% (95% CI 7.9%, 9.3), respectively (*P* < 0.001). The overall age and gender-standardized prevalence of polypharmacy was 16.4% (95% CI: 14.5%, 17.6%) and the estimated age-standardized prevalence of polypharmacy was 9.5% (95% CI: 7.5%, 11.6%) for males and 19.3% (95% CI: 17.2%, 21.4%) for females in GERD patients.

**Table 1:** Prevalence of polypharmacy, and Characteristics of patients with and without GERD enrolled in the Pars cohort study

	Patients with GERD		Patients without GERD		
Characteristics*	n (%)	Polypharmacy n (P%; 95% CI)	n (%)	Polypharmacy n (P%; 95% CI)	*P*-value^**^
**Overall**	2,325 (100)	365 (15.6; 14.2–17.2)	6,937 (100)	599 (8.6; 7.9–9.3)	< 0.001
**Gender**					
Male	843 (36.3)	73 (8.6; 6.7–10.5)	3432 (49.5)	131 (3.8; 3.1–4.4)	0.491
Female	1482 (63.7)	292 (19.7; 17.6–21.7)	3505 (50.5)	468 (13.3; 12.2–14.4)	
P	< 0.001		< 0.001		
**Age (years)**					
< 50	970 (41.7)	124 (12.7; 10.6–14.8)	3239 (46.7)	188 (5.8; 4.9–6.6)	0.253
50–59	679 (29.2)	96 (14.1; 11.5–16.7)	2146 (30.9)	206 (9.5; 8.3–10.8)	
≥ 60	676 (29.1)	145 (21.4; 18.3–24.5)	1552 (22.4)	205 (13.2; 11.5–14.8)	
P	0.001		0.001		
**Education**					
Literate	1304 (56.1)	216 (16.5; 14.5–18.5)	3234 (46.6)	348 (10.7; 9.6–11.8)	0.741
Illiterate	1021 (43.9)	149 (14.5; 12.4–16.7)	3703 (53.4)	251 (6.7; 5.9–7.5)	
P	0.195		< 0.001		
**Marital Status**					
Not married	342 (14.7)	61 (17.8; 14.1–22.2)	708 (10.2)	86 (12.0; 9.8–14.6)	0.294
Married	1,983 (85.3)	304 (15.3; 13.8–16.9)	6,229 (89.8)	513 (8.2; 7.5–8.9)	
P	0.239		0.001		
**Ethnicity**					
Fars	1278 (55.0)	228 (17.8; 15.7–19.9)	3937 (56.8)	399 (10.1; 9.1–11.0)	0.190
Non-Fars	1047 (45.0)	137 (13.0; 11.0- 15.1)	3000 (43.2)	200 (6.6; 5.7–7.5)	
P	0.002		< 0.001		
**Socio- Economic status**					
Low	725 (31.2)	101 (13.9; 11.4–16.4)	1694 (24.4)	108 (6.3; 5.2–7.5)	0.003
Low- Middle	614 (26.4)	99(16.1; 13.2–19.0)	1885 (27.2)	166 (8.8; 7.5–10.0)	
Middle-High	460 (19.8)	80 (17.3; 13.9–20.8)	1585 (22.8)	144 (9.0; 7.6–10.5)	
High	526 (22.6)	85 (16.1; 13.0- 19.3)	1773 (25.6)	181 (10.2; 8.7–11.6)	
P	0.418		0.001		
**Physical activity**					
Low	776 (33.4)	163 (21.0; 18.1–23.8)	2284 (32.9)	308 (13.4; 12.0- 14.8)	0.124
Moderate	773 (33.2)	131 (16.9; 14.3–19.5)	2283 (32.9)	191 (8.3; 7.2–9.5)	
High	776 (33.4)	71 (9.1; 7.1–11.1)	2370 (34.2)	100 (4.2; 3.4- 5.0)	
P	< 0.001		< 0.001		
**Body mass index (kg/m**^**2**^)					
< 25	955 (41.1)	117 (12.2; 10.1–14.3)	3145 (45.3)	173 (5.5; 4.7–6.2)	0.574
25–30	909 (39.1)	143 (15.7; 13.3–18.0)	2533 (36.5)	243 (9.5; 8.4–10.7)	
> 30	461 (19.8)	105 (22.7; 18.9–26.6)	1259 (18.2)	183 (14.5; 12.5–16.4)	
P	< 0.001		< 0.001		
**Metabolic syndrome**					
No	1387 (59.7)	138 (9.9; 8.3–11.5)	4592 (66.2)	180 (3.9; 3.3–4.4)	0.062
Yes	938 (40.3)	227 (24.2; 21.5–26.9)	2345 (33.8)	419 (17.8; 16.3–19.4)	
P	< 0.001		< 0.001		
**Alcohol use**					
No	2277 (97.9)	361 (15.8; 14.3–17.3)	6789 (97.9)	594 (8.7; 8.0- 9.4)	0.683
Yes	48 (2.1)	4 (8.3; 0.5, − 16.1)	148 (2.1)	5 (3.3; 1.4–7.8)	
P	0.156		0.021		
**Cigarette smoking**					
No	1920 (82.6)	323 (16.8; 15.1–18.4)	5425 (78.2)	542 (9.9; 9.1–10.7)	0.323
Yes	405 (17.4)	42 (10.3; 7.4–13.3)	1512 (21.8)	57 (3.7; 2.8–4.7)	
P	0.001		< 0.001		
**Tobacco use**					
No	1311 (56.4)	191 (14.5; 12.6–16.4)	4405 (63.5)	299 (6.7; 6.0- 7.5)	0.481
Yes	1014 (43.6)	174 (17.0; 14.7–19.4)	2532 (36.5)	300 (11.8; 10.5–13.1)	
P	0.090		< 0.001		
**Opium use**					
No	2118 (91.1)	343 (16.1; 14.6–17.7)	6370 (91.8)	580 (9.1; 8.3–9.8)	0.033
Yes	207 (8.9)	22 (10.6; 6.4–14.8)	567 (8.2)	19 (3.3; 1.8–4.8)	
P	0.036		< 0.001		
**No. of Comorbidities**					
0	0	0	1798 (25.9)	18 (1.0; 0.6–1.6)	< 0.001
1	292 (12.6)	12 (4.1; 2.1- 7.0)	1933 (27.9)	52 (2.7; 2.0- 3.5)	
2–3	1048 (45.1)	81 (7.7; 6.2–9.5)	2230 (32.1)	231 (10.4; 9.1–11.7)	
≥ 4	985 (42.3)	272 (27.6; 24.8–30.5)	976 (14.1)	298 (30.5; 27.6–33.5)	
P	0.001		< 0.001		

Table 1 provides the complete details of the characteristics of individuals with and without GERD in the Pars Cohort Study. Also, the number of concurrently used medications in GERD and non-GERD groups was significantly different and higher among patients with GERD (Fig. S1).

As shown in Table 2, among the factors associated with the prevalence of polypharmacy in GERD patients, having more than three comorbidities had the strongest association with the higher prevalence of polypharmacy (Adjusted PR: 3.33; 95% CI: 2.66, 4.15), while having high physical activity could be a protective factor (Adjusted PR: 0.69; 95% CI: 0.54, 0.88).

**Table 2:** Factors associated with the prevalence of polypharmacy among individuals with gastroesophageal reflux disease (GERD)

Factor		Crude Prevalence Ratio (95% CI)	Adjusted Prevalence Ratio (95% CI)	*P*-value
Current age (y) (Ref: <60)	≥ 60	1.79 (1.57, 2.05)	1.46 (1.19, 1.79)	< 0.001
Education (Ref: illiterate)	Literate	0.68 (0.59, 0.77)	1.43 (1.15, 1.78)	0.001
Gender (Ref: Male)	Female	3.19 (2.73, 3.72)	1.68 (1.30, 2.18)	< 0.001
Marital status (Ref: not married)	Married	0.71 (0.59, 0.85)	1.45 (1.14, 1.84)	0.002
Ethnicity (Ref: being non-Fars)	Fars	1.44 (1.26, 1.64)	1.25 (1.04, 1.50)	0.017
Metabolic syndrome (Ref: not having metabolic syndrome)	Yes	3.70 (3.23, 4.23)	1.77 (1.45, 2.15)	< 0.001
Physical activity (Ref: not having high level of physical activity)	High	0.41 (0.35, 0.49)	0.69 (0.54, 0.88)	0.004
Comorbidities (Ref: having less than 4 comorbidities)	≥ 4	4.23 (3.72, 4.82)	3.33 (2.66, 4.15)	< 0.001

We calculated the percentage of people with polypharmacy among individuals carrying the top nine underlying diseases, categorized according to GERD status (Fig. S2). Among individuals without GERD, polypharmacy was most common in those with heart disease (36.8%), diabetes mellitus (29.67%), and obstructive lung disease (22.08%). Similarly, among individuals with GERD, polypharmacy predominantly affected those with heart disease (42.9%), diabetes mellitus (38.82%), and, unlike the former group, depressive disorder (28.8%). Notably, the prevalence of polypharmacy was higher in individuals with GERD than those without GERD across eight out of the nine most common underlying diseases.

Table 3 shows the most common drug categories used by GERD patients in this study. Alimentary tract and metabolism (65.2%) and cardiovascular system (50.7%) drugs were the most prevalent drug categories used by men and patients older than 60. Among women, agents acting on the genitourinary system and sex hormones (65.6%) were the most common drugs, followed by agents acting on the alimentary tract and metabolism (52.8%). Moreover, according to Table 4, the drugs for acid-related disorders (A02 drug class) were the most prevalent alimentary tract and metabolism drugs used by total GERD patients (39.6%), men (39.3%), women (39.7%) and elders (45.5%).

**Table 3:** Anatomical Therapeutic Chemical classification of drugs used by individuals with gastroesophageal reflux disease in this study, n (%)

Drug class	Total	Men	Women	60 Years or Older
Alimentary tract and metabolism	1055 (58.0)	360 (71.6)	695 (52.8)	359 (65.2)
Genitourinary system and sex hormones	877 (48.2)	14 (2.8)	863 (65.6)	169 (30.7)
Cardiovascular system	623 (34.2)	163 (32.4)	460 (34.9)	279 (50.72)
Blood and blood-forming agents	450 (24.7)	65 (12.9)	385 (29.2)	116 (21.1)
Nervous systems	340 (18.7)	99 (19.68)	241 (18.3)	101 (18.4)
Musculoskeletal system	266 (14.6)	79 (15.7)	187 (14.2)	142 (25.8)
Other drugs	266 (14.6)	67 (13.3)	174 (13.2)	79 (14.4)

**Table 4:** The second ATC Classification level Alimentary tract and metabolism drugs besides Anti-inflammatory and antirheumatic products used by patients with GERD

Drug class	Total n = 2325 (100%)	Men n (%; 95% CI)	Women n (%; 95% CI)	60 years and older n (%; 95% CI)
A02	921 (39.6)	332 (39.3; 36.1–42.7)	589 (39.7; 37.2–42.2)	308 (45.5; 41.7–49.3)
M01	253 (10.8)	77 (9.1; 7.4–11.2)	176 (11.8; 10.3–13.6)	138 (20.4; 17.4–23.4)
A10	140 (6.0)	28 (3.32; 2.3–4.7)	112 (7.5; 6.3- 9.0)	66 (9.7; 7.6–11.9)
A11	66 (2.8)	3 (0.35; 0.2–1.1)	63 (4.25; 3.33–5.4)	20 (3.0; 1.9–4.5)
Aot	38 (1.63)	17 (2.0; 1.3–3.3)	21 (1.4; 1.0-2.2)	9 (1.3; 0.7–2.6)
A06	16 (0.7)	9 (1.0; 0.5- 2.0)	7 (0.4; 0.2–0.9)	8 (1.1; 0.5–2.3)
A12	14 (0.6)	2 (0.23; 0.1- 1.0)	12 (0.8; 0.5–1.5)	5 (0.7; 0.3–1.7)

Abbreviations: ATC: Anatomical Therapeutic Chemical; A02: Drugs for acid related disorders; M01: Anti-inflammatory and antirheumatic products; A10: Drugs used in diabetes; A11: Vitamins; Aot: other Alimentary tract and metabolism; A12: Mineral supplements blockers; A06: Drugs for constipation

**Fig. 1:**
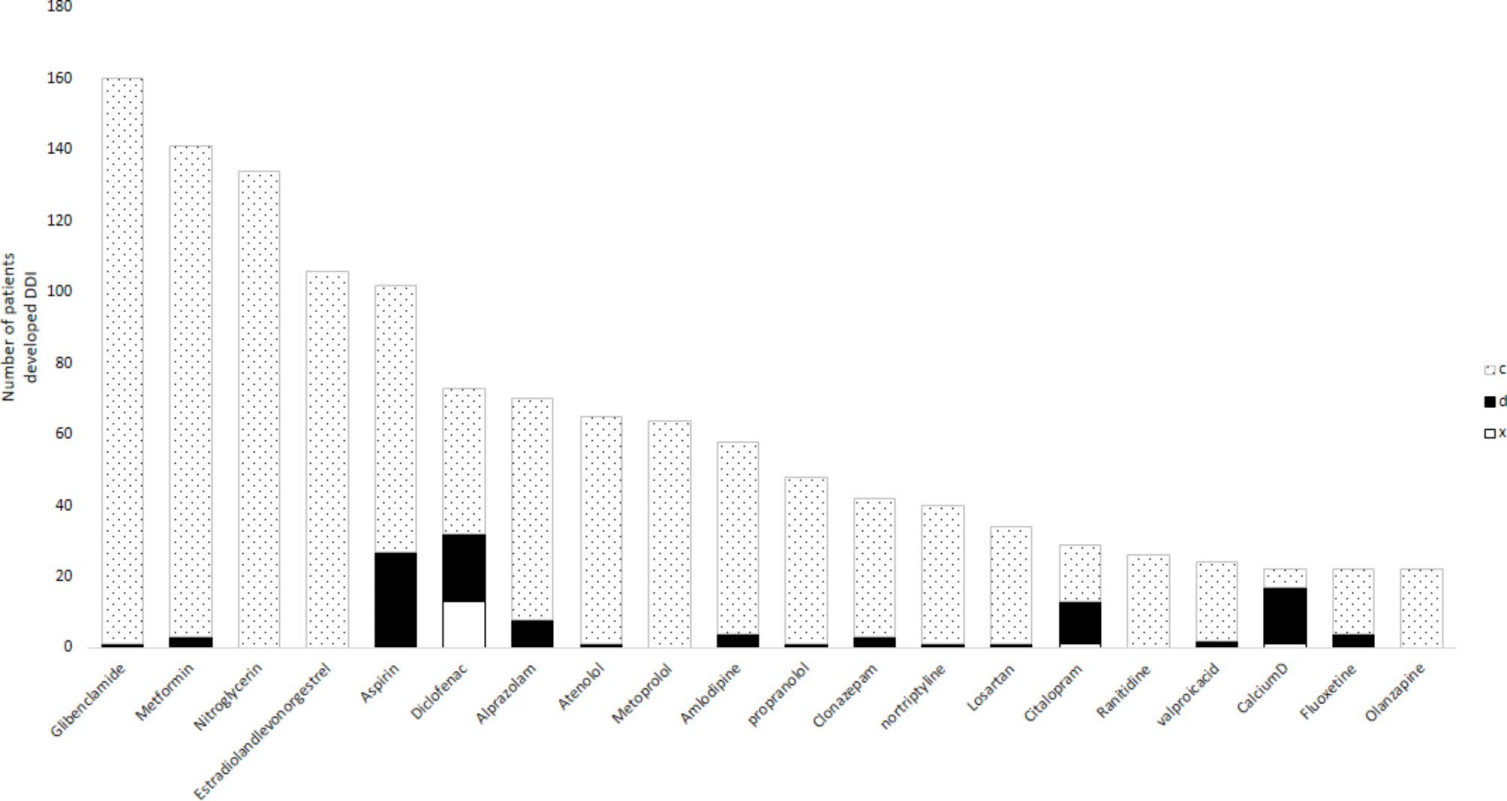
Most common drugs involved in potential drug-drug interactions in GERD patient

We found that 13.9%, 4.2%, and 1.1% of GERD patients had type C, D, and X drug-drug interactions, respectively. Glibenclamide/metformin, acetaminophen/ibuprofen, and diclofenac/ibuprofen were the most prevalent type C, D, and X drug-drug interactions among GERD patients, respectively. Metoprolol/nitroglycerine and Estradiol & levonorgestrel/Metformin were the second and third most prevalent type C interactions, respectively. Furthermore, the second and third type D drug-drug interactions were Acetaminophen/Methadone and Alprazolam/Tramadol, respectively. In terms of type X interactions, both Diclofenac/Naproxen and Diclofenac/piroxicam were tied for the second position, and Diclofenac/celecoxib held the third position (Fig. 1).

## Discussion

In this population-based study, we recorded the patterns of polypharmacy in individuals with GERD. Our results showed that among female patients, the prevalence of polypharmacy was almost twice that of males. Having more than three comorbidities, metabolic syndrome, the female gender, being more than 60 years old, being illiterate, being married, and being of the Fars ethnicity were factors associated with a higher prevalence of polypharmacy among GERD patients, while high physical activity was a protective factor.

A study conducted by Al-Dahshan et al. [24]. to explore the prevalence of polypharmacy among patients with non-communicable diseases showed higher polypharmacy prevalence (79.1%) in GERD patients. This study also showed the greater prevalence of polypharmacy among females which was in consistent with our results [26].

Central obesity, family history, smoking, tobacco use, hiatal hernia, impaired lower esophageal sphincter tone, delayed gastric emptying, and metabolic syndrome are some of the risk factors for GERD [27–30]. Moreover, some foods and drinks like green tea, alcohol, and coffee can induce GERD [31]. Some drugs may induce GERD or increase its symptoms. Zeynel Mungan et al. conducted a review to determine the role of drugs in GERD [32]. They reported that non-steroidal anti-inflammatory drugs (NSAIDs) are strongly associated with GERD. In addition, acetylsalicylic acid may increase the risk of GERD, especially if combined with NSAIDs. Estrogen-based hormone replacement therapy (HRT) and tricyclic antidepressants are also risk factors for GERD development, while anticholinergic drugs increase reflux episodes. Some cardiovascular drugs such as calcium channel blockers (CCBs) and nitrates can lead to GERD and worsen its symptoms. As it was revealed in this study, some drugs such as diclofenac and aspirin used by GERD patients were the most common drugs involved in type X and D interactions. These drugs also can contribute to GERD symptoms exacerbation. So physicians should take note of the different medications that individuals with GERD are using.

We found that polypharmacy was more prevalent among female patients. This can be attributed to the fact that women usually pay more attention to their health status than men and tend to consume more drugs and seek more health services [33]. In addition, self-medication by women in the absence of rigid prescription guidelines and drug availability, especially in Iran, causes excessive drug use and so, polypharmacy [34].

In this study, we found that comorbidities were associated with a higher risk of polypharmacy. In fact, polypharmacy was consistently more common in individuals with GERD compared with those without GERD for eight out of the nine most common comorbidities. One reason is that each disease requires specific medications prescribed by different specialists, with GERD itself sometimes requiring multiple medications. We must note that variations in medications prescribed by different physicians for different diseases can cause adverse drug reactions [35, 36]. Using polypills instead of multiple drugs is one way to reduce this complication [37]. It is also suggested that physicians check the patients’ drug lists and consult with the related specialists. Additionally, polypharmacy itself can be a risk factor for having more than one disease as some drugs may cause certain complications and illnesses. Therefore, polypharmacy and multimorbidity could make a vicious cycle. This relation should be considered in future studies.

A strong bidirectional association exists between physical function and polypharmacy: better physical function is associated with a lower risk of polypharmacy, while polypharmacy is associated with lower physical function [38]. In our work, we found that physical activity could protect against polypharmacy in individuals with GERD. This could root in the more proper health behaviors as a result of higher physical activity in individuals who are physically active compared with those who have less physical activity [39]. In line with our findings, one study in Germany found that increased physical activity reduced the risk of polypharmacy among multimorbid persons aged 65 and older [40]. Hence, interventions to promote physical activity may have immense population health benefits, one mechanism of which appears to be through minimizing the rate of polypharmacy.

In this study, GERD patients’ most common drug classes were, in order, the drugs for alimentary tract and metabolism, genitourinary system and sex hormones, cardiovascular system, blood and blood-forming agents, nervous systems, and musculoskeletal system. No other study has classified the various medications used by individuals with GERD. Among Alimentary tract and metabolism drugs, we showed that drugs for acid related disorders were the most used drugs taken by men, women and elders; However, we found that more than half of GERD patients were not taking drugs for acid-related disorders. These agents can lead to a variety of drug-drug interactions [41]. Hence, in approaching a patient with GERD symptoms, physicians should be aware of common medications they may be using.

This study was the first to specifically explore the link between polypharmacy and GERD. One of the limitations of this study was that although trained medical doctors were utilized to assess medical history and physical examination, cardiac chest pain still may not be completely differentiated from heartburn. Another limitation was this study’s cross-sectional nature. Due to the burden of GERD on patients and healthcare systems and considering the adverse impacts of polypharmacy, further studies on this topic are warranted.

## Conclusion

Our population-based study indicates that polypharmacy is more prevalent in individuals with GERD than those without GERD, meaning that patients with GERD should be monitored carefully in terms of their drug lists. Among patients with GERD, those with comorbidities, females, and those with metabolic syndrome are at higher risk of polypharmacy, so the healthcare system should provide due care. On the other hand, physical activity might protect against polypharmacy in GERD patients. Considering the fairly high rate of potential drug-drug interactions identified, a review of the medication list is essential when approaching such patients, and physicians must check for medications that may worsen GERD, particularly calcium channel blockers, nitrates, and NSAIDs. Besides confirmatory studies in different settings, researchers should also look into interventions to minimize polypharmacy’s burden on individuals with GERD, particularly among the mentioned high-risk subgroups.

### Electronic supplementary material

Below is the link to the electronic supplementary material.

**Supplementary Material 1: Supplementary Figure 1**. The number of concurrently used drugs in the groups with and without gastroesophageal reflux disease (GERD)

**Supplementary Material 2: Supplementary Figure 2**. Prevalence of polypharmacy among individuals carrying the top nine underlying diseases, categorized according to gastroesophageal reflux disease (GERD) status

## Data Availability

The data are available from the corresponding author upon reasonable request.
