# Supplementary material for: Polypharmacy and pattern of medication use among patients with gastroesophageal reflux disease: results from Pars Cohort study
Source: BMC Gastroenterol. 2023 Dec 14;23:439. doi: 10.1186/s12876-023-03086-7 (PMC10720105; doi:10.1186/s12876-023-03086-7)
Supplement: Supplementary file 1 — Supplementary Material 1: Supplementary Figure 1. The number of concurrently used drugs in the groups with and without gastroesophageal reflux disease (GERD) [file 12876_2023_3086_MOESM1_ESM.docx]

**Supplementary Figure 1.** The number of concurrently used drugs in the groups with and without gastroesophageal reflux disease (GERD).
