# Supplementary material for: Polypharmacy and pattern of medication use among patients with gastroesophageal reflux disease: results from Pars Cohort study
Source: BMC Gastroenterol. 2023 Dec 14;23:439. doi: 10.1186/s12876-023-03086-7 (PMC10720105; doi:10.1186/s12876-023-03086-7)
Supplement: Supplementary file 2 — Supplementary Material 2: Supplementary Figure 2. Prevalence of polypharmacy among individuals carrying the top nine underlying diseases, categorized according to gastroesophageal reflux disease (GERD) status [file 12876_2023_3086_MOESM2_ESM.docx]

**Supplementary Figure 2.** Prevalence of polypharmacy among individuals carrying the top nine underlying diseases, categorized according to gastroesophageal reflux disease (GERD) status
